# Study of the mechanism by gentiopicroside protects against skin fibroblast glycation damage via the RAGE pathway

**DOI:** 10.1038/s41598-024-55525-4

**Published:** 2024-02-26

**Authors:** Chunyu Chen, Xiaoxing Liu, Li Li, Miaomiao Guo, Yifan He, Yinmao Dong, Hong Meng, Fan Yi

**Affiliations:** 1 Beijing Key Laboratory of Plant Resources Research and Development, Beijing Technology and Business University, No. 11, Fucheng Road, Haidian District, Beijing, 100048 People’s Republic of China; 2 The School of Light Industry Science and Technology, Beijing Technology and Business University, No. 11, Fucheng Road, Haidian District, Beijing, 100048 People’s Republic of China

**Keywords:** Gentiopicroside, Glycosylation, AGE inhibitor, RAGE, Drug delivery, Pharmacology, Cell adhesion, Cell growth, Cell migration, Organelles, Senescence

## Abstract

The occurrence of nonenzymatic glycosylation reactions in skin fibroblasts can lead to severe impairment of skin health. To investigate the protective effects of the major functional ingredient from Gentianaceae, gentiopicroside (GPS) on fibroblasts, network pharmacology was used to analyse the potential pathways and targets underlying the effects of GPS on skin. At the biochemical and cellular levels, we examined the inhibitory effect of GPS on AGEs, the regulation by GPS of key ECM proteins and vimentin, the damage caused by GPS to the mitochondrial membrane potential and the modulation by GPS of inflammatory factors such as matrix metalloproteinases (MMP-2, MMP-9), reactive oxygen species (ROS), and IL-6 via the RAGE/NF-κB pathway. The results showed that GPS can inhibit AGE-induced damage to the dermis via multiple pathways. The results of biochemical and cellular experiments showed that GPS can strongly inhibit AGE production. Conversely, GPS can block AGE-induced oxidative stress and inflammatory responses in skin cells by disrupting AGE-RAGE signalling, maintain the balance of ECM synthesis and catabolism, and alleviate AGE-induced dysfunctions in cellular behaviour. This study provides a theoretical basis for the use of GPS as an AGE inhibitor to improve skin health and alleviate the damage caused by glycosylation, showing its potential application value in the field of skin care.

## Introduction

Advanced glycosylation end products (AGEs) are cross-linked substances formed at the late stage of the glycosylation reaction between glucose (or other reducing sugars) and free amino groups in proteins, nucleic acids, or lipids; these substances, the existence of which was first proposed by the French chemist Maillard in 1912, are brown in colour and are fluorescent^1^. In 1981, Monnier and Cerami^2^ discovered that the glycosylation reaction is related to biological processes. The accelerated ageing of skin mesenchymal tissues and collagen under the influence of nonenzymatic browning has generated widespread concern. Comprehensive studies have found that the production of AGEs is irreversible and that AGEs are not easily metabolized. The deterioration of the natural environment and the modern high-sugar diet have led to higher rates of accumulation of AGEs in the dermis layer of the skin, which results in structural disorders, functional degradation, and skin abnormalities, further leading to various types of health problems^3^.

AGEs regulate gene expression by binding to the cell surface receptor RAGE, which activates the transcriptional activation of NF-κB, leading to the expression of many inflammatory factors, such as the inflammatory cytokines interleukin 6 (IL-6) and interleukin 1β (IL-1β), which results in the development of skin inflammatory responses^4,5^. AGEs and RAGE also induce the overexpression of ROS, which trigger oxidative stress and damage the mitochondrial membrane potential^6^. The cascading effects of oxidative stress and inflammatory eruption lead to continuous accumulation of AGEs in the skin, forming a positive feedback loop^7^.

Conversely, AGEs contribute to the overexpression of MMP-2 and MMP-9 and the breakdown of collagen (COL-1), fibronectin (FN-1), and laminin (LN-5) in the extracellular matrix, leading to the disruption of the balance between synthesis and degradation of the extracellular matrix (ECM) and impaired and destabilized ECM remodelling. Disruption of the cellular microenvironment leads to an inability to maintain the biological behaviours of dermal cells (proliferation, migration, and adhesion)^8^, with adverse effects on the structure and function of skin. Recent studies have proposed a new perspective on skin ageing^9–11^, wherein the interaction of the ECM and human dermal fibroblasts (HDFs) is highly correlated with skin ageing. The ECM provides mechanical strength, elasticity, and an environment that supports the biological functions of fibroblasts. Disruption of the dynamic balance of the ECM usually results in the generation of an ageing fibroblast phenotype, marked by the collapse of cellular morphology, loss of contraction and extension, and loss of ability to secrete a new ECM. Concurrently, the ECM is unable to adequately regulate cellular processes, including cell proliferation, migration, and adhesion.

Cellular behavioural dysfunction, oxidative stress, and inflammatory factor overexpression due to glycosylation reactions are important causes of structural disorders, functional degradation, and abnormalities in the skin^12,13^. Therefore, AGE inhibitors are a key means of improving the skin health of the population, and the development of AGE inhibitors to protect the normal functioning of skin cells is an important goal of current research.

Gentiopicroside (GPS) is the main active ingredient present in species belonging to Gentianaceae. It is a cleaved-ring enol ether terpene glycoside with the chemical formula C_16_H_20_O_9_ and presents as white or yellowish acicular crystals under normal conditions. AGEs are highly correlated with the disease progression of diabetes, and GPS has been previously studied in relation to diabetes mellitus. GPS can effectively improve glucose-lipid metabolism disorders and renal dysfunction by regulation of the CK2/NF-κB inflammatory signalling pathway via AT1R^14^ and can improve cardiac function in diabetic rats by targeting Smad3 phosphorylation to attenuate high glucose-induced inflammation, oxidative stress, and activation of cardiac fibroblasts^15^. The excellent anti-inflammatory and antioxidative stress pharmacological effects of GPS make it a potential inhibitor of AGEs. However, the antiglycosylation effect of GPS on skin has not yet been clarified, and its effect on human skin as an AGE inhibitor and the structural basis of this effect have not yet been elucidated, so further in-depth studies are needed to understand the stages of its biological effects and the underlying mechanisms.

The present study first used network pharmacological analysis to assess the potential of GPS to modulate glycosylation-related signalling pathways to ameliorate skin glycosylation injury. Biochemical and cellular experiments were designed to further investigate the inhibitory effects of GPS on glycosylation and to analyse its potential inhibitory mechanisms. A skin model of methylglyoxal (MGO)-induced glycosylation injury was established to investigate the ameliorative effect of GPS on cellular behavioural dysfunction and related mechanisms, as well as its role in alleviating cellular oxidative stress and inflammation based on the modulation of the RAGE receptor. This study provides a theoretical basis for the use of GPS as an AGE inhibitor to improve the treatment of glycosylation-related skin lesions and improve the skin health of the population and shows its potential application value in the skincare field.

## Results and discussion

### GPS has potential as an inhibitor of AGEs

In this study, we first applied network pharmacology to explore the pharmacological relevance and potential molecular mechanisms underlying the antiglycosylation activity of GPS on skin. Based on network pharmacology, we identified the shared genes from among the GPS target genes and skin-related genes, explored the potential of GPS to ameliorate skin glycosylation damage and the related signalling pathways by GO functional enrichment analysis and KEGG pathway analysis, and preliminarily assessed whether this is a plausible basis for the exploration of the antiglycosylation activity of GPS.

**Figure 1:**
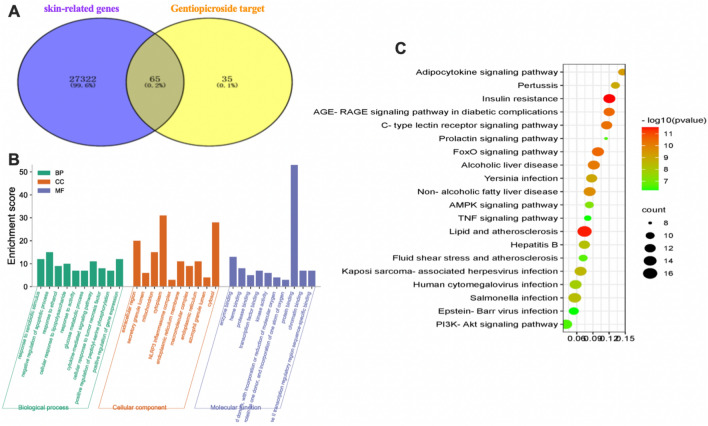
Network pharmacology investigation exploring the pharmacological mechanisms underlying the action of GPS on skin. (**A**) Intersection of GPS targets and skin-related genes. (**B**) Results of GO functional enrichment analysis. (**C**) Results of KEGG pathway enrichment analysis.

The 100 experimentally validated gene targets of GPS in the PubChem database were compared with 27,387 skin-related genes in the GeneCards database, and 65 overlapping genes were identified. The match rate was 65%, which indicated that the gene targets regulated by GPS were highly correlated with the skin genes and that GPS was therefore closely associated with skin (Fig. 1A).

The results of GO functional enrichment analysis showed that the skin-related genes targeted by GPS were mainly involved in pathways related to the following GO terms: the GO terms comprised 182 biological processes (BPs), such as “response to xenobiotic stimulus”, “negative regulation of apoptotic process”, “response to ethanol”, “cellular response to lipopolysaccharide”, “enzyme binding”, “response to lipopolysaccharide” and “glucose metabolic process”; 21 cellular components (CCs), such as “extracellular region” and “secretory granule lumen”; and 40 molecular functions (MFs), such as “enzyme binding” and “transcription factor binding.” The top 10 entries in the MF, BP, and CC categories were filtered according to the P value (Fig. 1B).

The results of KEGG pathway analysis (https://www.kegg.jp/kegg/kegg1.html) showed that the GPS targets related to skin were enriched in a total of 134 pathways, including cellular senescence, endocrine system, and viral infection, and they were significantly related to the AGE/RAGE glycosylation-related signalling pathway and the FOXO inflammation-related signalling pathway (Fig. 1C). The raw data are detailed in the Supplementary 1.

Based on the results of network pharmacology, GPS may have the potential to alleviate skin glycosylation damage by regulating the AGE/RAGE signalling pathway. The participation of GPS in the BPs of “glucose metabolism” and “regulation of oxidoreductase activity” suggests that GPS has the potential to be used as an AGE inhibitor, and our preliminary assessment is that GPS can be considered a pilot substance with antiglycosylation activity for further investigation.

### GPS inhibits the formation of AGEs at both the biochemical and cellular levels

Based on the results of the web-based pharmacological prediction, the inhibitory effect of GPS on glycosylation was verified at the biochemical and cellular levels.

BSA contains 583 amino acid residues, with many acidic and basic amino acids on the surface, a certain amount of arginine and lysine, and high stability, which makes it a good protein for use as an in vitro glycosylation substrate. By constructing a BSA-fructose reaction solution incubation model to simulate the process of nonenzymatic glycosylation of proteins in vivo and detecting the amount of fluorescent AGEs generated in the model, the inhibitory effect of GPS on nonenzymatic glycosylation of proteins can be explored at the biochemical level. Liu et al.^17^ detected the inhibitory effect of *Rosmarinus officinalis* glycoside extract on the fluorescence of fluorescent AGEs by building a BSA-glucose model. Kumagai et al.^18^ investigated the inhibitory effect of pomegranate extract on glycosylation by using a BSA-sugar (glucose, fructose) reaction model.

**Figure 2:**
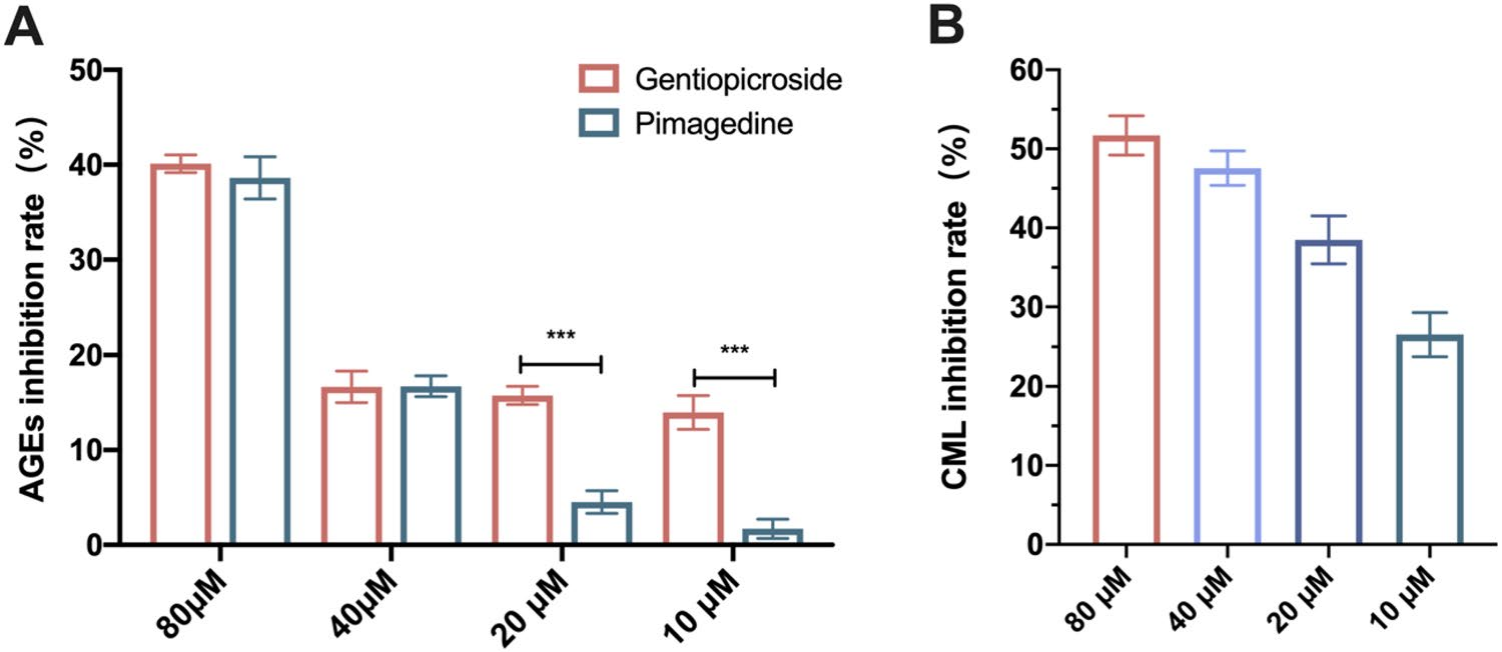
Inhibition of AGEs by GPS at the biochemical and cellular levels in vitro. (**A**) Inhibition of AGEs by different concentrations of GPS. (**B**) Inhibition of CML by different concentrations of GPS.

GPS strongly inhibited fluorescent AGEs in a concentration-dependent manner (Fig. 2A). The AGE inhibition rate of GPS at a concentration of 80 μM (28 μg/mL) was 40.12%, and the inhibition rates at concentrations of 20 μM and 10 μM were significantly higher than those in the AG positive control group at the same concentrations (16.66% and 13.96%, respectively). The experimental results indicated that GPS showed excellent antiglycosylation activity in the biochemical assay.

MGO, a highly reactive dicarbonyl compound formed during glycosylation, is the structural precursor of CML, one of the most prevalent AGEs present in skin, and is an excellent inducer of AGEs^3^. Studies by Guillon et al.^19^ and others similarly demonstrated that carbonyl compounds are excellent for the study of AGEs associated with glycosylation-related skin complications and are excellent cellular models. AGEs mainly attack dermal skin cells; therefore, in this study, we used MGO to treat skin fibroblasts (CCC-ESF-1 cells) to simulate the accumulation of AGEs in human skin and explored the inhibitory effect of GPS on the nonenzymatic glycosylation of proteins at the cellular level by detecting changes in the CML content.

A CCC-ESF-1 cell model with 500 μM MGO-induced glycosylation injury was used to determine the CML content after the addition of 80 μM, 40 μM, 20 μM, or 10 μM GPS. As shown in Fig. 2B, GPS significantly reduced the CML content in glycosylated cells in a concentration-dependent manner. The CML inhibition rate of GPS was as high as 51.70% at a concentration of 80 μM, and GPS still had a 26.52% inhibition rate at a concentration of 10 μM. The excellent ability of GPS to inhibit the glycosylation reaction was further verified at the cellular level.

In conclusion, the results from in vitro biochemical experiments and cellular experiments showed that GPS has excellent antiglycosylation efficacy in skin and is an excellent inhibitor of AGEs.

### GPS ameliorates MGO-induced cell behavioural dysfunction by restoring ECM protein expression

Multiple studies have shown that AGEs tend to accumulate in the ECM of the dermis and regulate the overexpression of the ECM-related genes MMP-2 and MMP-9 in fibroblasts, leading to a disruption in the balance between synthesis and degradation of the ECM. MMP-2 and MMP-9 are matrix metalloproteinases that degrade collagen isoforms, fibronectin (FN-1), and laminin (LM-5); MMP-9 overexpression disrupts the ECM and weakens cell adhesion and migration. The deleterious effect of glycosylation on the ECM destroys the microenvironment for cell survival and ultimately leads to impaired cell behaviour (proliferation, migration, and adhesion)^8^.

**Figure 3:**
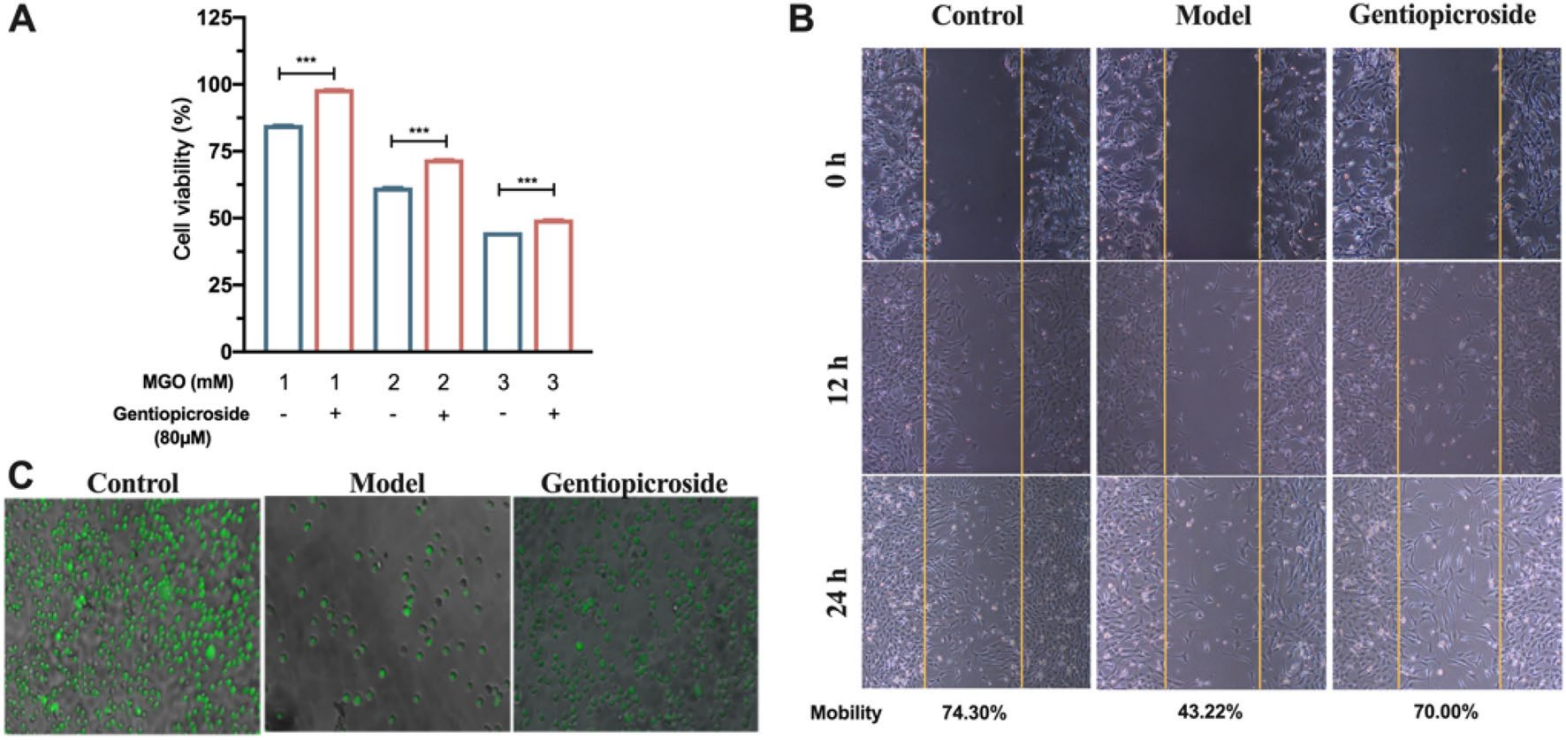
Abnormal cell behavioural function improved by GPS. (**A**) Restoration of cell proliferation ability by different concentrations of GPS. (**B**) Restoration of cell migration ability by different concentrations of GPS. (**C**) Restoration of cell migration adhesion ability by different concentrations of GPS.

The GPS concentration with optimal antiglycosylation efficacy (80 μM) was used as the experimental concentration to investigate the effect of GPS on MGO-induced cell viability under stimulation with different concentrations of MGO. The cell viability under stimulation with 1, 2, or 3 mM MGO decreased gradually with increasing concentration, and the cell viability of the treatment groups under stimulation with the same concentrations of MGO was significantly enhanced (*P* < 0.001), with proliferation rates of 13.38%, 13.38%, 10.80%, 4.80%, 4.80%, 10.54%, and 4.80%. The experimental results indicated that GPS antagonized MGO-induced cell damage (Fig. 3A).

Cell migration is important for ECM remodelling and maintaining the normal state and structure of the skin, so we used a scratch assay to detect cell migration ability. GPS (80 μM) improved the dysfunction of cell migration caused by glycosylation injury. The migration rates (scratch repair rate) of the blank control group, MGO group, and treatment + MGO group were 74.30%, 43.33%, and 63.15%, respectively (Fig. 3B). Compared with MGO, the 80 μM treatment + MGO significantly increased the cell migration rate by approximately 19.93%, restoring the MGO-induced cell migration dysfunction to a near-normal level.

Changes in cell adhesion were detected using a cell adhesion assay with glycosylated matrix protein. As shown in Fig. 3C, the number of wall-adherent cells in the MGO-treated group was significantly reduced, and the 80 μM gentiopicroside + MGO treatment restored the MGO-induced cell adhesion dysfunction to a small extent. The adhesion rate was 71.51% in the blank control group, 56.37% in the MGO-treated group, and 62.18% in the 80 μM gentiopicroside-treated group, which restored approximately 6% of the cell adhesion function. The experimental results showed that GPS restored MGO-induced cell adhesion dysfunction to a small extent.

GPS antagonized the cellular damage induced by MGO and improved the viability and proliferation of skin cells undergoing glycosylation injury. MGO induced impaired skin cell migration and adhesion, which was similar to the findings of ChunTao Yang et al.^20^, and GPS restored the abnormalities in cellular behavioural functions to a certain extent.

FN-1 enhances cell migration and proliferation by helping to maintain the cytoskeleton, and LM-5 is a major component of the cell basement membrane that stimulates cell adhesion and cell movement during cell development. Vimentin protein plays a role in cellular morphological integrity and skeleton stability and maintains cell contraction function; therefore, in this study, we investigated the mechanisms related to the improvement of glycosylation-induced behavioural dysfunction in fibroblasts by detecting changes in the expression of the important constituent proteins of the ECM, namely, FN-1, LM-5, COL-1, and vimentin, caused by GPS.

**Figure 4:**
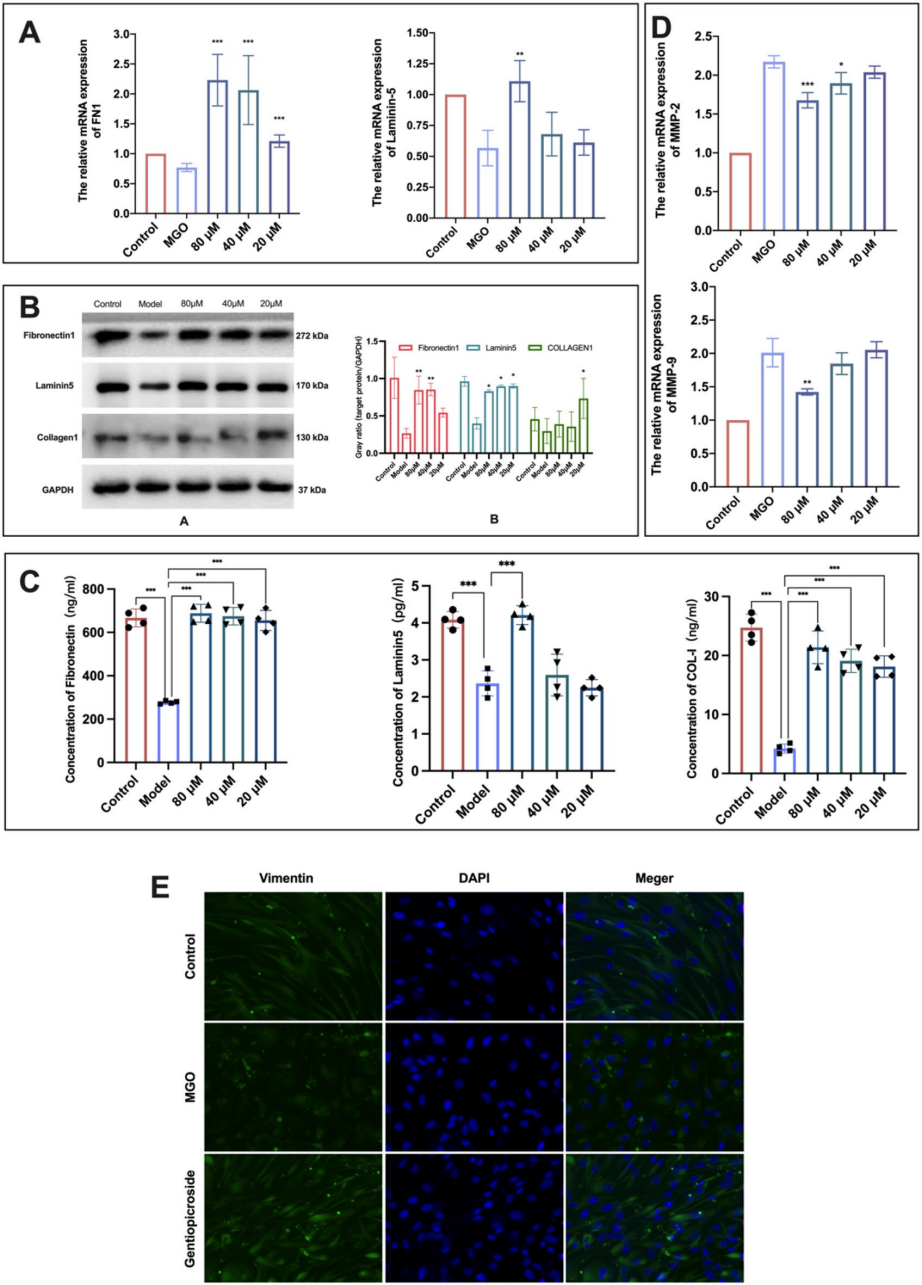
GPS regulates the synthesis of ECM proteins and regulates the expression of vimentin. (**A**) qRT-PCR assay to detect the expression of ECM protein mRNAs. (**B**) WB assay to detect the expression of ECM proteins. (**C**) ELISA to detect the expression of ECM proteins. (**D**) qRT-PCR assay to detect the expression of MMP-2 and MMP-9 mRNAs. (**E**) Immunofluorescence assay to detect vimentin protein expression. All the target investigated genes and housekeeping genes were counted on the same WB membrane for statistical examination.

qRT‒PCR was used to detect the effects of the treatments on the regulation of the gene expression levels of FN-1 and LM-5 based on the glycosylation cell model (Fig. 4A). At concentrations of 80 μM, 40 μM, and 20 μM, GPS significantly upregulated (*P* < 0.001) the relative expression of FN-1 mRNA, and 80 μM GPS significantly increased (*P* < 0.01) the relative expression of LM-5 mRNA. Western blot analysis further verified the improvement of key ECM protein levels by GPS (Fig. 4B). The Western blot results further verified that gentiopicroside had an ameliorative effect on the levels of key ECM proteins (Fig. 4B). Compared with the blank control group, treatment with MGO significantly reduced the levels of FN-1, LM-5, and COL-1 in the ECM. The GPS + MGO treatment significantly restored the decrease in ECM protein content caused by MGO. The raw data are detailed in the Supplementary 2.

Changes in the levels of FN-1, LM-5 and COL-1 in the ECM were detected by ELISA to further confirm that GPS has an ameliorative effect on the levels of key proteins in the ECM (Fig. 4C). The levels of FN-1, LM-5, and COL-1 were significantly decreased with MGO-induced injury, with values of 277.51 ng/mL, 2.37 pg/mL, and 4.22 ng/mL, respectively. The ECM protein content with MGO injury was significantly restored (*P* < 0.001) by treatment with 80 μM GPS, with values of 688.20 ng/mL, 4.21 pg/mL, and 21.38 ng/mL and recovery rates of 61.59%, 44.99%, and 69.4%, respectively, all of which were restored to the normal levels.

MMP-2 and MMP-9 are gelatinases, and the main substrates degraded by gelatinases are gelatine, fibronectin (FN-1), laminin (LM-5), and elastin in basement membranes, as well as various types of collagen. Overexpression of MMP-9 destroys the ECM and attenuates cell adhesion and migration. qRT-PCR was used to detect the role of GPS in regulating the gene expression levels of MMP-2 and MMP-9 based on the glycosylation cell model. The experimental results are shown in Fig. 4D. The relative expression of MMP-2 and MMP-9 mRNA increased significantly with MGO induction, and GPS downregulated the relative expression of MMP-2 and MMP-9 mRNA.

Vimentin plays a role in cellular morphological integrity and skeletal stability and maintains cell contractile function, which plays an important role in wound healing and the ageing process. Immunofluorescence staining was used to detect the distribution and structural status of vimentin. The control group had a patterned (scale-like, neatly arranged) normal wide distribution. With MGO-induced glycosylation damage, vimentin was redistributed to the nuclear periphery of the aggregation, the organization was altered, and the structure of vimentin was changed. The results were similar to the findings of Kueper et al.^21^. However, 80 μM GPS partially restored the organization and structure of vimentin and maintained the scale-like cell arrangement (Fig. 4E).

Under conditions of high aggregation of AGEs, the skin’s ability to resist exogenous damage is significantly reduced due to microscopic changes in histology and cellular function, which ultimately leads to the occurrence of structural disorders of the skin, susceptibility to infections, and prolonged healing of injuries. GPS may downregulate the expression of MMPs and restore the ECM protein content under MGO stimulation to maintain the microenvironment of cell survival, laying an important foundation for the normal functions of cell proliferation, migration and adhesion.

### GPS ameliorates cellular oxidative stress, and the inflammatory response is based on the modulation of RAGE receptors

AGEs induce oxidative stress by binding to the cell surface receptor RAGE, which induces the overexpression of ROS. ROS are the most important inducers of the inflammatory response, and oxidative stress is also an important factor in the formation of endogenous AGEs. When an organism is under long-term oxidative stress, the body’s innate defence mechanisms are exhausted, leading to the overproduction and accumulation of ROS, which ultimately results in the massive production of AGEs, forming a positive feedback loop^5,7^. Concurrently, AGEs regulate gene expression by binding to the cell surface receptor RAGE, which activates the transcriptional activation of NF-κB in a series of cascading reactions, leading to the expression of many inflammatory factors, such as the inflammatory cytokines interleukin 6 (IL-6) and interleukin 1β (IL-1β), resulting in inflammatory skin responses^4,5^.

Therefore, reducing oxidative stress due to glycosylation and decreasing the overexpression of inflammatory factors are important means to ameliorate skin cell damage caused by the induction of glycosylation^22,23^.

**Figure 5:**
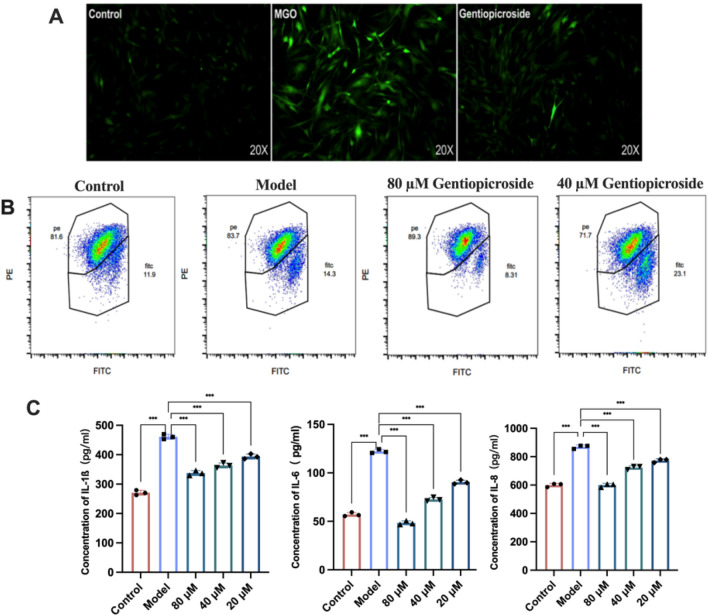
GPS modulates the cellular oxidative stress status and inflammatory response. (**A**) Fluorescence detection of cellular ROS levels. (**B**) Flow cytometry detection of cellular mitochondrial membrane potential damage. (**C**) ELISA detection of inflammatory factor expression.

The experimental results showed that after MGO stimulation, the intracellular ROS content increased, and the fluorescence intensity was significantly enhanced. GPS (80 μM) significantly reduced the intracellular ROS content and effectively reduced the oxidative stress induced by glycosylation in cells (Fig. 5A).

Mitochondria are major targets of oxidative stress. The continuous accumulation of ROS in cells triggers mitochondrial dysfunction, which is an early indicator of skin cell damage. Flow cytometry was used to detect the ameliorative effect of *Gentianaceae* bitter glycosides on mitochondrial membrane potential damage based on glycosylation reactions. When JC-1 was present as a monomer in the cells, green fluorescence (FITC), low MMP, and mitochondrial dysfunction were observed. As shown in Fig. 5B, the experimental results showed that green fluorescence was significantly enhanced in the model group (23.1%) compared with the normal group (8.30%), and the mitochondrial membrane potential was reduced by MGO treatment to 11.9% and 14.3% in the 80 μM and 40 μM treatment groups, respectively, suggesting that GPS may significantly ameliorate MGO-induced damage to the mitochondrial membrane potential by lowering the ROS content, thereby protecting normal mitochondrial function.

MGO induces the release of a large amount of inflammatory factors by mediating the AGE/RAGE/NF-κB signalling pathway, and an excessive inflammatory response is a major reason for the nonhealing of glycosylated skin wounds and structural tissue abnormalities. ELISA was used to detect the release of intracellular IL-6, IL-8, and IL-1β and to observe the effect of GPS on the inflammatory factor content in MGO-induced skin cells. As shown in Fig. 5C, the experimental results indicated that the relative content of inflammatory factors increased significantly under MGO stimulation, and 80 μM, 40 μM and 20 μM GPS can significantly reduce (*P* < 0.001) the production of IL-6, IL-8 and IL-1β, effectively alleviated the inflammatory response of glycosylated skin cells.

In conclusion, GPS could reduce the intracellular ROS content, alleviate glycosylation-induced oxidative stress, and significantly improve glycosylation-induced mitochondrial membrane potential damage. It also significantly reduced the expression levels of inflammatory factors, thus improving the inflammatory response of glycosylated skin cells.

The effects of AGEs on skin are usually triggered by physiological and pathological responses that are modulated by the RAGE receptor. The binding of AGEs to RAGE induces intracellular oxidative stress, which stimulates the upregulation of ROS and the sustained activation of NF-κB, leading to the production of large amounts of inflammatory factors downstream. This sustained activation induces the expression of RAGE and further exacerbates the production of endogenous AGEs (Bierhaus et al.^7^), thus forming a positive feedback loop.

The present study proposes that GPS may ameliorate oxidative stress and inflammatory responses induced by fibroblasts due to glycosylation by modulating the AGE/RAGE/NF-κB signalling pathway and thereby ameliorating the oxidative stress and inflammatory responses induced by fibroblasts.

**Figure 6:**
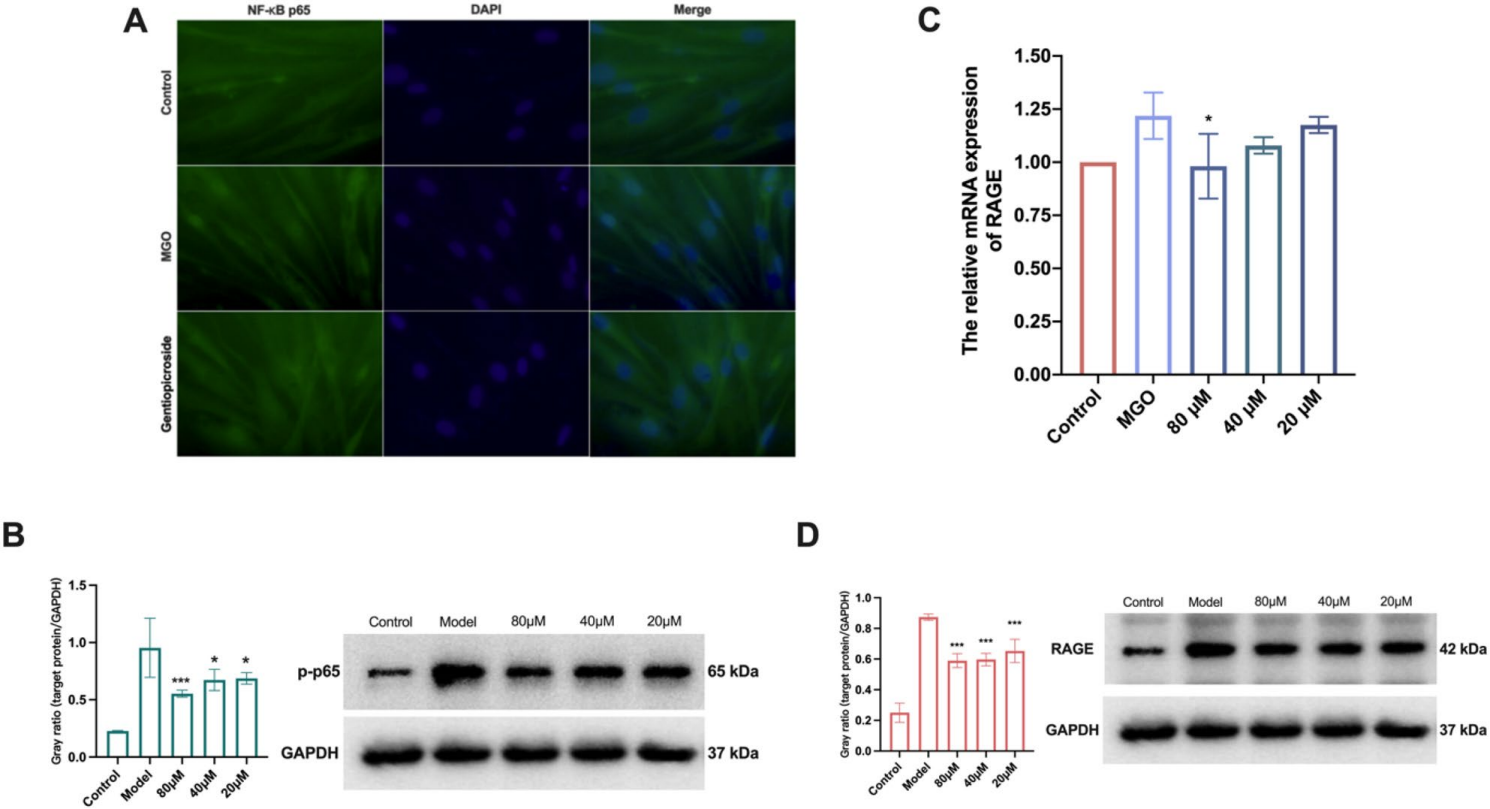
Examination of the RAGE/NF-κB pathway regulated by GPS. (**A**) Immunofluorescence examining the expression of NF-κB p65. (**B**) Western blot examining the expression of NF-κB p65. (**C**) qRT-PCR examining the expression of RAGE. (**D**) Western blot examining the expression of RAGE receptor. All the target investigated genes and housekeeping genes were counted on the same WB membrane for statistical examination.

First, immunofluorescence staining was used to observe the intracellular distribution status of NF-κB p65 and explore the regulation of NF-κB activation by GPS. As shown in Fig. 6A, the experimental results showed that NF-κB p65 was normally distributed in the cytoplasm in the blank control group, NF-κB p65 was phosphorylated and shifted to the nucleus in the MGO group, and the degree of nuclear displacement of this protein could be attenuated by GPS. The effects of different concentrations of GPS on phosphorylated NF-κB p65 protein levels were detected by Western blotting using GAPDH as an internal reference protein, and the results are shown in Fig. 6B. The MGO-induced increase in the expression level of the p-p65 protein and the activation of the NF-κB signalling pathway were observed, and the phosphorylation of NF-κB p65 was significantly downregulated (*P* < 0.001) by 80 µM GPS treatment, which thus blocked the activation. The results of both experiments showed that MGO could promote the activation of NF-κB, while GPS could inhibit the MGO-induced activation of NF-κB.

qRT‒PCR was used to detect the effect of GPS on RAGE receptor transcript levels. As shown in Fig. 6C, the experimental results indicated that the expression of RAGE mRNA increased under MGO stimulation, and GPS at 80 μM significantly reduced (*P* < 0.05) the expression of RAGE under MGO stimulation. Western blotting was used to detect the effects of MGO and GPS on RAGE expression. As shown in Fig. 6D, compared with the blank control group, the MGO-treated group exhibited significantly upregulated expression of RAGE, and GPS (80, 40, and 20 μM) significantly decreased (*P* < 0.001) the expression of RAGE. The experimental results indicated that GPS decreased the expression of inflammatory factors by mediating the AGE/RAGE/NF-κB signalling pathway and thus the expression of inflammatory factors. The raw data are detailed in the Supplementary 2.

Based on the above experimental results, GPS can alleviate glycosylation-induced oxidative stress and inflammatory responses, and these effects are achieved by inhibiting RAGE expression. GPS ameliorates cellular damage in fibroblasts due to glycosylation by modulating the AGE/RAGE/NF-κB signalling pathway and thus ameliorating cellular damage in fibroblasts.

## Conclusion

Because AGEs are irreversibly formed and not easily metabolized, under the deteriorating living conditions and modern high-sugar diets of various populations, AGEs tend to accumulate in large quantities in the dermal layer of the skin, resulting in structural disorders, functional degradation and abnormalities of the skin and triggering various types of skin health problems. In this study, we investigated the antiglycosylation efficacy of GPS and its protective effect on human dermal fibroblasts damaged by glycosylation as an inhibitor of AGEs and the related mechanisms.

The present study first used network pharmacological analysis to assess the potential of GPS to modulate relevant signalling pathways to ameliorate skin glycosylation injury. Biochemical and cellular experiments were designed to investigate the inhibitory effect of GPS on glycosylation and to analyse its potential inhibitory mechanism. A skin model of MGO-induced glycosylation injury was established to investigate the amelioration of cellular behavioural dysfunction by GPS and its related mechanisms, as well as the alleviation of cellular oxidative stress and inflammation based on the modulation of the RAGE receptor.

**Figure 7:**
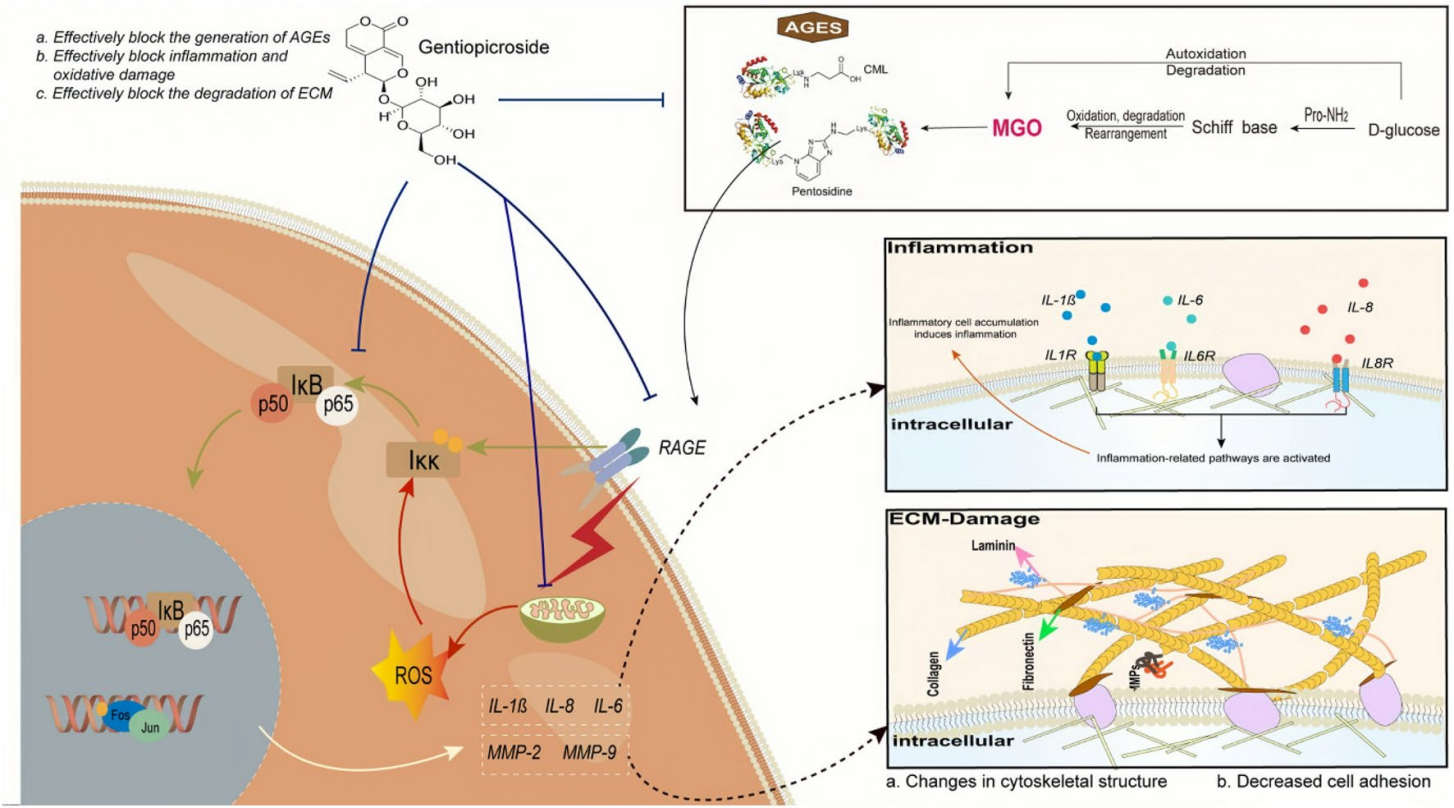
GPS could inhibit AGE-induced damage to the dermis via multiple pathways.

The results showed that GPS could inhibit AGE-induced damage to the dermis via multiple pathways (Fig. 7). The results of biochemical and cellular experiments showed that GPS could strongly inhibit AGE formation. Conversely, GPS also blocked the damage to skin cells caused by AGEs after their formation. First, GPS blocked the oxidative stress and inflammatory response of skin cells caused by AGEs by disrupting AGE/RAGE axis signalling. Second, GPS improved cell behavioural dysfunction caused by AGEs by maintaining the balance of ECM synthesis and catabolism. This study provides a theoretical basis for the use of GPS as an AGE inhibitor to improve skin health and alleviate the damage caused by glycosylation and shows its potential application value in the field of skin care.

## Materials and methods

### Network construction for GPS target genes and comparison of common genes associated with GPS and skin

Genes experimentally validated as being associated with GPS use were compiled from the PubChem (PubChem; https://pubchem.ncbi.nlm.nih.gov/) database and used to construct the GPS gene collection. Genes related to skin were collected using the GeneCards database (GeneCard; http://www.genecards.org/). The 100 genes in the GPS gene collection were compared with the 27,387 genes in the skin gene collection to identify overlapping genes. A network was constructed using the overlapping genes to assess the interactions between GPS and skin.

### Functional enrichment analysis

Gene Ontology (GO) functional enrichment analysis and Kyoto Encyclopedia of Genes and Genomes (KEGG) pathway analysis were performed on the overlapping target genes using DAVID (http://david.ncifcrf.gov). The species was limited to humans, and the relationships between the GPS gene set and the skin gene set were predicted based on potential molecular functions and molecular interactions. The enrichment results were visualized using the Microbiotics website (http://www.bioinformatics.com.cn/).

### *Establishment of an *in vitro* nonenzymatic glycosylation incubation system*

Based on the method of Spagnuolo et al.^16^, bovine serum albumin (BSA) solution (20 mg/mL) and fructose solution (0.5 moL/L) were prepared with 0.2 M phosphate-buffered saline (PBS, pH = 7.2–7.4, 0.02% sodium azide as a preservative), filtered through a 0.45 μm aqueous filter membrane, and then mixed in a 1:1 equal volume to obtain the BSA-fructose reaction solution.

$${\text{IR}}\left( {\text{\% }} \right) = 1 - \frac{{{\text{RFU}}_{{{\text{inhibition}}\,{\text{group}}}} - {\text{RFU}}_{{{\text{blank}}\,{\text{group}}}} }}{{{\text{RFU}}_{{{\text{negative}}\,\,{\text{control}}\,\,{\text{group}}}} }} \times 100\%$$

### Quantification of the glycosylation end product (CML)

CCC-ESF-1 cells were cultured with MGO to stimulate the production of large amounts of AGEs and to establish a glycosylated skin cell model with high expression of AGEs. CCC-ESF-1 cells were stimulated according to the conditions for the MGO-induced glycosylation injury cell model, and diluted GPS solution was added simultaneously. After 48 h of incubation at 37 ℃ with 5% CO_2_, the cell lysates were obtained, and the carboxymenthyl lysine (CML) content was measured with the Human Carboxymethyl Lysine CML ELISA Kit.

### CCK-8 assay

CCC-ESF-1 cells were seeded in 96-well plates at a density of 1 × 10^4^ cells/well, and each group had 3–6 replicate wells. Cells were cultured overnight for adhesion, and different intervening factors were added after medium replacement. The treatment concentration for optimal antiglycosylation efficacy was found to be 80 μM, and this was used as the experimental concentration. The experimental treatment groups were set as follows: 1 mM MGO + 80 μM GPS, 1 mM MGO + serum-free medium, 2 mM MGO + 80 μM GPS, 2 mM MGO + serum-free medium, 3 mM MGO + 80 μM GPS, and 3 mM MGO + serum-free medium, with serum-free medium (DMEM) used as a blank control. After 48 h of incubation, the supernatant was aspirated and discarded, 100 µL of CCK-8 working solution was added, and the OD at 450 nm (OD_450_) was measured. The cell viability was calculated according to the following formula:

$${\text{Cell}}\,{\text{viability}}\left( \% \right) = \frac{{{\text{OD}}_{{{\text{sample}}}} }}{{{\text{OD}}_{{{\text{control}}}} }} \times 100\%$$

### Scratching test

CCC-ESF-1 cells were seeded in 6-well plates at a density of 5 × 10^5^ cells/well, and the confluency reached 100% after overnight culture. After aspirating the culture solution, the cells were scored vertically with a 10 μL pipette tip. Different treatments were added before incubation. The cells were placed in an incubator and then removed at 0, 12, and 24 h of incubation. After imaging under a microscope, the area of no cell growth and the total area were calculated using ImageJ software.

### Cell adhesion assay

Cell adhesion was measured using the Cell Adhesion Assay Kit (BB-48123, Bestbio). One hundred microlitres of inclusion solution was added to each well of a 96-well plate, and the plate was incubated at 4 °C overnight. After removing the inclusion solution, the 96-well plate was air-dried and washed three times with washing solution. Cells pretreated with GPS and MGO in a 6-well plate were digested with 0.25% trypsin, centrifuged, resuspended in fresh serum-free medium, and seeded at 5 × 10^3^ cells/well in the pretreated 96-well plate. The plates were placed in the incubator for 2 h, the medium was discarded, and the plates were then washed twice. Then, 200 µL of basal medium and 10 µL of BBcell Probe C07 fluorescence staining solution were added to each well, and cell adhesion was observed and measured using an inverted fluorescence microscope and a luciferase marker with an excitation wavelength of 488 nm and an emission wavelength of 520 nm.

### ELISA for ECM-associated proteins

CCC-ESF-1 cells were collected at the end of the MGO and treatment incubations, and the cell supernatant was obtained. The levels of fibronectin, laminin-5, and type I collagen in the supernatant were determined using ELISA kits (CLOUD-CLONE) according to the manufacturer’s instructions.

### ROS detection

In this experiment, a reactive oxygen species (ROS) detection kit was used. After cells plated in 6-well plates had completed the treatment incubations and were washed with PBS, 2 mL of dichloro-dihydro-fluorescein diacetate (DCFH-DA; 10 µM) diluted with basal medium was added to each well, and incubation was continued for 20 min. The basal medium was washed 2 to 3 times, 1 mL of PBS was added, and the fluorescence of the cells was observed and photographed under an inverted fluorescence microscope.

### Mitochondrial membrane potential assay

In this experiment, loading of the JC-1 probe was performed according to the manufacturer’s instructions for the mitochondrial membrane potential assay kit. Cells plated in six-well plates were scraped with a cell scraper, and the cell suspension was collected in centrifuge tubes. The supernatant was discarded, and the cell pellet was collected after centrifugation at 1000 rpm for 5 min. The cells were added to 0.5 mL of basal medium and 0.5 mL of JC-1 stain working buffer; the contents of the tubes were mixed by inversion. After homogenization, the cells were incubated for 20 min and centrifuged at 1000 rpm for 5 min; the supernatant was then aspirated and discarded. The cells were washed with JC-1 staining buffer 2–3 times and resuspended in JC-1 staining buffer, and the fluorescence was detected by flow cytometry using an excitation wavelength of 488 nm and the FL1 (Em = 525 ± 20 nm) and FL2 (Em = 585 ± 20 nm) fluorescence channels for detection. The results were analysed using Flow Jo software.

### Detection of the inflammatory factors IL-6, IL-8 and IL-1β by ELISA

At the end of cell incubation with MGO and the treatments, cell supernatants were collected. The levels of the three inflammatory factors in the supernatant were determined using the Human IL-6 ELISA Kit, Human IL-1ß ELISA Kit and Human TNF-α ELISA Kit (Solarbio). The specific steps are detailed in the manufacturer’s instructions.

### Immunofluorescence staining assay

At the end of treatment incubation, cells were fixed in 4% paraformaldehyde, permeabilized with 0.3% Triton X-100 and blocked with 1% BSA. They were then incubated with primary antibodies (an anti-vimentin antibody [ab8978] and anti-NF-kB p65 antibody [ab16502]) and secondary antibodies, stained with DAPI for visualization of nuclei, and then observed under a microscope.

### qRT‒PCR

At the end of treatment incubation, total RNA was extracted from cells with TRIzol (total RNA extraction reagent), and cDNA was synthesized using the First Strand cDNA Synthesis Kit (containing double-stranded DNAse) and further quantified by real-time fluorescence using Fast Super EvaGreen^®^ qPCR Master Mix. The primers used in the experiment were designed and compared in the NCBI database, as shown in Table 1.

**Table 1:** Primer sequences.

Primers		Sequence
β-actin	F	CGCGAGAAGATGACCCAGAT
	R	GCACTGTGTTGGCGTACAGG
Laminin-5	F	GGACTGCAGGCCACCG
	R	AGGATGCCCACAAACTCCAG
FN1	F	TCAGCTTCCTGGCACTTCTG
	R	TCTTGTCCTACATTCGGCGG
MMP-2	F	GTCTGTGTTGTCCAGAGGCA
	R	CTAGGCCAGCTGGTTGGTTC
MMP-9	F	CTTTGAGTCCGGTGGACGAT
	R	TCGCCAGTACTTCCCATCCT

### Western blotting assay

At the end of treatment incubation, cells were collected and lysed with RIPA lysis buffer (containing 1% PMSF), the protein concentration was measured with a BCA protein content determination kit, and 5 × loading buffer was prepared and mixed with the samples at a 4:1 volumetric ratio. The samples were placed in a dry bath at 100 °C for 5 min and then cooled on ice. The samples were resolved by SDS‒PAGE, followed by transfer to a PVDF membrane using a rapid transfer system. The membrane was blocked with 5% BSA after transfer. Exposure and imaging were performed in a chemiluminescent image analysis system after incubation with primary antibodies (FN1, LM5, COL-I, RAGE and p-p65) and secondary antibodies. Greyscale analysis of the bands was performed using ImageJ software. All the target investigated genes and housekeeping genes were counted on the same WB membrane for statistical examination.

### Statistics and analysis

The data in this paper were obtained from three or more parallel experiments and are expressed as the mean ± standard deviation. Data visualization and statistical analysis were performed using GraphPad Prism 9 and ImageJ, difference analysis was performed by the One-way ANOVA method, and *P* < 0.05 was considered a statistically significant difference.

### Supplementary Information

Supplementary Information 1.

Supplementary Information 2.

## Data Availability

The raw data related to network pharmacology and WB presented in this study are detailed in the Supplementary Information. Data supporting the results of this study are available from the corresponding author upon reasonable request.
