# Supplementary material for: Study of the mechanism by gentiopicroside protects against skin fibroblast glycation damage via the RAGE pathway
Source: Sci Rep. 2024 Feb 26;14:4685. doi: 10.1038/s41598-024-55525-4 (PMC10897486; doi:10.1038/s41598-024-55525-4)
Supplement: Supplementary file 2 — Supplementary Information 2. [file 41598_2024_55525_MOESM2_ESM.docx]

**COL1-130kDa**

**Control**

**Model**

**80μmol GPS**

**40μmol GPS**

**20μmol GPS**

**FN1-272kDa**

**Control**

**Model**

**80μmol GPS**

**40μmol GPS**

**20μmol GPS**

**LM5-170kDa**

**Control**

**Model**

**80μmol GPS**

**40μmol GPS**

**20μmol GPS**

**RAGE-42kDa**

**Control**

**Model**

**80μmol GPS**

**40μmol GPS**

**20μmol GPS**

**p65-65kDa**

**Control**

**Model**

**80μmol GPS**

**40μmol GPS**

**20μmol GPS**

**GAPDH-37kDa**

**Control**

**Model**

**80μmol GPS**

**40μmol GPS**

**20μmol GPS**
